# Ethnicity-specific blood pressure thresholds based on cardiovascular and renal complications: a prospective study in the UK Biobank

**DOI:** 10.1186/s12916-024-03259-5

**Published:** 2024-02-05

**Authors:** Donghan Su, Huanhuan Yang, Zekun Chen, Yuhao Kong, Xiaona Na, Queran Lin, Ai Zhao, Yan Zheng, Yanan Ma, Xiaoyu Li, Zhihui Li

**Affiliations:** 1 Vanke School of Public Health, Tsinghua University, Beijing, China; 2 Clinical Research Design Division, Sun Yat-Sen Memorial Hospital, Sun Yat-Sen University, Guangzhou, China; 3 Breast Tumor Center, Sun Yat-Sen Memorial Hospital, Sun Yat-Sen University, Guangzhou, China; 4 Institute for Healthy China, Tsinghua University, Beijing, China; 5 State Key Laboratory of Genetic Engineering, School of Life Sciences, Fudan University, Shanghai, China; 6 Department of Biostatistics and Epidemiology, School of Public Health, China Medical University, Shenyang, China; 7 Department of Sociology, Tsinghua University, Beijing, China; 8 Department of Social and Behavioral Sciences, Harvard T.H. Chan School of Public Health, Boston, MA USA

**Keywords:** Hypertension thresholds, Ethnicity, Cardiometabolic complications, Renal complications

## Abstract

**Background:**

The appropriateness of hypertension thresholds for triggering action to prevent cardiovascular and renal complications among non-White populations in the UK is subject to question. Our objective was to establish ethnicity-specific systolic blood pressure (SBP) cutoffs for ethnic minority populations and assess the efficacy of these ethnicity-specific cutoffs in predicting adverse outcomes.

**Methods:**

We analyzed data from UK Biobank, which included 444,418 participants from White, South Asian, Black Caribbean, and Black African populations with no history of cardiorenal complications. We fitted Poisson regression models with continuous SBP and ethnic groups, using Whites as the referent category, for the composite outcome of atherosclerotic cardiovascular disease, heart failure, and chronic kidney disease. We determined ethnicity-specific thresholds equivalent to the risks observed in Whites at SBP levels of 120, 130, and 140 mm Hg. We adjusted models for clinical characteristics, sociodemographic factors, and behavioral factors. The performance of ethnicity-specific thresholds for predicting adverse outcomes and associated population-attributable fraction (PAF) was assessed in ethnic minority groups.

**Results:**

After a median follow-up of 12.5 years (interquartile range, 11.7–13.2), 32,662 (7.4%) participants had incident composite outcomes. At any given SBP, the predicted incidence rate of the composite outcome was the highest for South Asians, followed by White, Black Caribbean, and Black African. For an equivalent risk of outcomes observed in the White population at an SBP level of 140 mm Hg, the SBP threshold was lower for South Asians (123 mm Hg) and higher for Black Caribbean (156 mm Hg) and Black African (165 mm Hg). Furthermore, hypertension defined by ethnicity-specific thresholds was a stronger predictor and resulted in a larger PAF for composite outcomes in South Asians (21.5% [95% CI, 2.4,36.9] vs. 11.3% [95% CI, 2.6,19.1]) and Black Africans (7.1% [95% CI, 0.2,14.0] vs. 5.7 [95% CI, -16.2,23.5]) compared to hypertension defined by guideline-recommended thresholds.

**Conclusions:**

Guideline-recommended blood pressure thresholds may overestimate risks for the Black population and underestimate risks for South Asians. Using ethnicity-specific SBP thresholds may improve risk estimation and optimize hypertension management toward the goal of eliminating ethnic disparities in cardiorenal complications.

**Supplementary Information:**

The online version contains supplementary material available at 10.1186/s12916-024-03259-5.

## Background

In the United Kingdom (UK), marked ethnic disparities in cardiometabolic diseases remain a major public health challenge. Mortality and morbidity rates from coronary heart disease (CHD) and stroke are 50–100% higher among South Asians compared to the White population [1–4]. The African and Caribbean population in the UK shows a 20–50% lower risk of CHD but a 1.5–2.5 times higher risk of stroke compared to the other ethnic groups [1–4], which is different from the USA.

High blood pressure (BP) is the most critical risk factor for cardiometabolic diseases, affecting one-fourth of the world’s adult population [5]. Observational studies consistently show that there is an independent and continuous association between blood pressure and the risk of hemorrhagic and ischaemic stroke, CHD, heart failure, and end-stage renal disease [6]. The 2023 European Society of Hypertension (ESH) guideline for the management of arterial hypertension defines grade 1 hypertension as office measurements of systolic blood pressure (SBP) ≥ 140 mm Hg and/or diastolic blood pressure (DBP) ≥ 90 mm Hg, irrespective of ethnicity [7].

The existing thresholds were established based on evidence from outcome-based randomized clinical trials demonstrating the benefits of treatments for patients with those blood pressure measurements [7]. However, most trials did not recruit sufficient participants from ethnic minority groups to estimate the treatment effects. For instance, the Systolic Hypertension in Europe (Syst-Eur) Trial did not report the ethnic composition of the participants, who were presumably predominantly White [8]. The Hypertension in Diabetes Study in the UK recruited less than 10% of participants from non-White populations [9]. Thus, there is limited evidence regarding the optimal BP targets and treatment modalities in ethnic subgroups [7]. It is important to note that the same BP level may lead to varying cardiometabolic risks among ethnic minority groups. For example, a study in the UK demonstrated persistent disparities in the risk of cardiometabolic diseases across different ethnic groups, even after adjusting for blood pressure, other conventional cardiovascular risk factors, and socioeconomic factors [4]. Furthermore, research conducted in the UK and the USA has identified a stronger association between systolic BP and stroke in South Asians [10] and African Americans [11] compared to Whites. Additionally, in the USA, the Black population has a higher prevalence and severity of hypertension-mediated organ damage compared to the White population, regardless of the BP levels [12]. Therefore, due to ethnic disparities in the absolute risk of cardiometabolic diseases and the differential strength of associations between blood pressure and its complications, the optimal threshold for defining hypertension and initiating blood pressure-lowering interventions may differ across various ethnic groups.

Several studies have attempted to derive ethnicity-specific BMI thresholds to address ethnic disparities in obesity and diabetes risk. These studies consistently reported lower BMI thresholds for obesity in ethnic minority populations [13–16]. However, it is concerning that similar efforts have yet to be made to establish ethnicity-specific blood pressure thresholds in relation to cardiovascular and renal complications. Most available cohorts that could potentially accomplish this task recruited small numbers of ethnic minority populations, resulting in issues of statistical power to derive ethnicity-specific thresholds. To bridge these knowledge gaps, we utilized the UK Biobank cohort, which has recruited a sufficient number of participants from Black Caribbean, Black African, and South Asian backgrounds. Our goal was to establish ethnicity-specific SBP thresholds that correspond to the risks of cardiovascular and renal complications associated with thresholds established in White populations. Additionally, we aimed to assess the performance of ethnicity-specific thresholds in predicting adverse cardiovascular and renal outcomes.

## Methods

### Study population and study design

This prospective study utilized data from the UK Biobank, a large cohort that recruited more than 500,000 participants aged 40–69 years from 22 assessment centers located in England, Scotland, and Wales between 2006 and 2010. During baseline visits, participants completed touch-screen questionnaires, underwent verbal interviews conducted by trained staff to gather health information, provided biological samples, and received anthropometric measurements after providing informed consent. For further details on this cohort’s study design and data collection methods, please refer to a previous publication [17].

Our study was restricted to participants who did not have prevalent chronic kidney disease and existing cardiovascular diseases such as myocardial infarction, stroke, and heart failure. We identified these conditions through the integration of self-reported medical history during the baseline interview and linkage to primary care and hospital inpatient records. We excluded participants with missing information on blood pressure, ethnicity, age, Townsend deprivation index, smoking status, and drinking status (less than 5% missingness). Age was derived based on the date of birth and date of attending an initial assessment, and thus, it could be missing due to a missing date of birth. Furthermore, we excluded participants who did not identify themselves as one of the four ethnic groups or mixed ethnicities: White, South Asian, Black Caribbean, and Black African. Eventually, 444,418 participants were included in the analysis. The participant inclusion and exclusion flow chart is presented in Additional file 1: Fig. S1.

### Primary predictors and covariates

Following a minimum 5-min period of participant rest while seated, trained nurses utilized an Omron digital HEM-705IT monitor to measure blood pressure twice, with a short interval between readings. The blood pressure measurements were taken in a consistent manner during the initial assessment visits that took place from 2006 to 2010. The device was validated for professional and home use in adults under the British Hypertension Society and the European Society of Hypertension criteria [18, 19]. We calculated the average of the two blood pressure measurements. Following the previous literature, we calculated blood pressure as the average of two manual blood pressure measurements if automated measurements were missing to increase our sample size [20, 21]. The proportion of participants with manual blood pressure measurements, the mean manual SBP measurements, and mean automated SBP measurements across ethnic groups were presented in Additional file 1: Table S1. Based on the current guidelines, we classified blood pressure as follows: optimal (SBP < 120 mm Hg and DBP < 80 mm Hg), normal (SBP 120–129 mm Hg and/or DBP 80–84 mm Hg), high normal (SBP 130–139 mm Hg and/or DBP 85–89 mm Hg), and grade 1 hypertension (SBP 140–159 mm Hg and/or DBP 90–99 mm Hg) [7]. Following previous practice [14, 22], we identified the participants' ethnicity based on their self-reports. During the baseline assessment, participants used a touch-screen questionnaire to self-report their ethnicity, choosing from six options: White, Mixed, Asian, Black, Chinese, or other ethnic groups. Asian includes Indian, Pakistani, Bangladeshi, or any other Asian background. Black includes Caribbean, African, and any other Black background. We categorized ethnicity as White, South Asian, Black Caribbean, and Black African to maximize statistical power.

Possible confounding factors included sociodemographic factors, lifestyle characteristics, clinical characteristics, and biomarkers. Participants self-reported sociodemographic information, clinical characteristics, and lifestyle factors through questionnaires or during a verbal interview at baseline. Sociodemographic factors included age, sex, education, and Townsend Deprivation Index. Townsend deprivation index was an area-level measure of material deprivation calculated from information on unemployment, non-car ownership, non-home ownership, and household overcrowding and divided into quintiles, with the fifth quintile representing the most deprived. Lifestyle characteristics included drinking status, smoking status, and physical activities. Smoking status and drinking status were categorized into current, former, and never. Physical activities were represented by total metabolic equivalent task minutes per week for all activities, including walking, moderate and vigorous activity [17]. Clinical characteristics included diabetes status, hypertension medication use, cholesterol-lowering medication use, and body mass index (BMI). BMI was calculated as weight in kilograms (measured using the Tanita BC 418 body composition analyzer) divided by the square of standing height in meters (measured using the SECA 240 height measure). We classified BMI as follows: < 18.5 kg/m^2^, 18.5–24.9 kg/m^2^, 25.0–29.9 kg/m^2^, and ≥ 30 kg/m^2^. Blood samples were collected at the baseline visit, and biomarkers were analyzed at the UK Biobank’s central laboratory. Biomarkers included low-density lipoprotein cholesterol (LDL-C), high-density lipoprotein cholesterol (HDL-C), triglycerides, and creatinine. We estimated the glomerular filtration rate (eGFR) using the 2021 creatinine-based Chronic Kidney Disease Epidemiology Collaboration equation without race [23]. We followed the updates in the National Institute of Health and Care Excellence (NICE) chronic kidney disease (CKD) guideline that removed the recommendation to adjust for the Black ethnicity due to concerns of overestimating the eGFR [24]. Urine microalbumin assays only covered 156,557 participants among all UK Biobank participants, so we did not include the albumin creatinine ratio in our analyses.

### Outcome ascertainment

Following the previous literature, we selected the study endpoint based on the most important complications of hypertension [25, 26], which included incident atherosclerotic cardiovascular disease (ASCVD), incident heart failure (HF), and CKD. We defined ASCVD as a composite of non-fatal myocardial infarction, non-fatal stroke, or death from cardiovascular causes. Further details on the specific conditions included in the outcome definition and the corresponding ICD-9/10 codes used for identifying them can be found in Additional file 1: Table S2. We ascertained incident ASCVD, CKD, and HF after baseline assessment through linkage to hospital inpatient records kept by Hospital Episode Statistics for England (censoring date 31 October 2022), Scottish Morbidity Record (censoring date 31 July 2021), and Patient Episode Database for Wales (censoring date 28 February 2018). We obtained information about the date and cause of death from death certificates that were kept by the National Health Service Information Centre in England and Wales, as well as the National Health Service Central Register Scotland, with a censoring date of 30 November 2022. More information about how the records were connected can be found at https://content.digital.nhs.uk/services.

### Statistical analyses

We summarized the baseline characteristics of each ethnic group using the mean or interquartile range for continuous variables and frequencies, along with percentages for categorical data.

As in previous studies [15, 27–30], we conducted a three-step process to identify SBP thresholds that are equivalent in risk to those observed in the White population. We did not derive ethnicity-specific thresholds for DBP, as DBP was not consistently associated with the risk of the composite outcome in all ethnicity populations, and previous studies have shown that SBP seems to be a superior predictor of adverse outcomes than DBP after midlife [5]. First, we fitted Poisson regression models using continuous SBP and all ethnic groups, with White as the reference category, for the composite outcome of atherosclerotic cardiovascular disease, heart failure, and chronic kidney disease. Models were adjusted for DBP (with splines at 65 mmHg), age, sex, BMI, income, education, drinking status, smoking status, diabetes status, hypertension medications, cholesterol-lowering medications, LDL-C, HDL-C, triglycerides, eGFR (with splines at 60 mL/min/1.73m^2^), Townsend deprivation index, and physical activities. To explore the potential effect of immigrant history, we additionally adjusted the model for the duration of residence in the UK (≥ 20 years or < 20 years). Participants who were born in the UK were considered to have lived in the UK for more than 20 years. We attempted to model SBP using restricted cubic splines with 3 knots and quadratic terms to see whether it could improve model fit. In the second step, we estimated the predicted incidence rates for European participants at SBP levels of 120, 130, and 140 mmHg. For South Asian, Caribbean, and African populations, we estimated predicted incidence rates for SBP levels ranging from the 5th to 95th percentiles with increments of 0.1 mmHg. In the third step, we identified the SBP levels for South Asian, Caribbean, and African populations that yielded the same incidence rate as the SBP thresholds for Whites. To account for the significant impact of age and sex on the relationship between blood pressure and outcomes, we repeated the same process and derived ethnicity-specific thresholds within each of these subgroups. Since blood pressure classifications are crucial for diagnosing hypertension in untreated populations and determining treatment targets in those receiving antihypertensive medication [31] and anti-hypertensive drugs may significantly impact the outcomes, we calculated the thresholds for subgroups defined by antihypertensive medication use.

We constructed Poisson regression models for each ethnicity subgroup to assess the performance of the ethnicity-specific SBP thresholds for predicting cardiovascular and renal complications. In these models, we included a binary variable for grade 1 hypertension defined by ethnicity-specific SBP thresholds vs. ESH-recommended thresholds and adjusted for the same covariates previously mentioned. We also calculated the population-attributable fraction of the composite outcome associated with grade 1 hypertension in each ethnicity group, which indicates the proportion of the composite outcome that could be eliminated by eliminating grade 1 hypertension as defined by ethnicity-specific thresholds instead of the guideline-recommended thresholds. Analyses were performed using Stata version 17.0 (StataCorp, LLC).

## Results

The analysis included 444,418 participants, with 428,929 (96.5%) being White, 8454 (1.9%) being South Asian, 3991 (0.9%) being Black Caribbean, and 3044 (0.7%) being Black African (Table 1). The average age of participants was 57.0 (interquartile range, 50.0–63.0), and all non-white participants were younger than the White participants. Black African had the highest average SBP (138.4 ± 18.5 mm Hg) and DBP (85.1 ± 10.7 mm Hg), while South Asian and Black Caribbean had lower average SBP (134.6 ± 18.4 mm Hg and 136.6 ± 18.5 mm Hg, respectively) than Whites (137.9 ± 18.6 mm Hg), but not for DBP. There are no clinically significant differences between the manual SBP measurements and the automated SBP measurements in each ethnic group (Additional file 1: Table S1). All non-white populations were more likely to have the lowest household income (26.4%, 26.8%, and 30.5% for South Asian, Black Caribbean, and Black African, respectively) and to be more deprived (36.8%, 56.0%, and 72.4% for South Asian, Black Caribbean, and Black African, respectively) than the White population (18.3% with the lowest household income and 17.6% most deprived). Compared to the White population (23.2% with obesity and 3.8% with diabetes), Black Caribbean and Black African were more likely to be obese (37.4% and 42.6%, respectively) and have diabetes (9.3% and 8.9%, respectively), and South Asian were more likely to have diabetes (13.7%) and use cholesterol-lowering medication (20.0%). Black Caribbean had the largest proportion of current smokers (16.7%), and South Asian and Black African had smaller proportions of current drinkers and smokers than Whites.

**Table 1:** Baseline characteristics of participants in UK biobank included in analyses by self-reported ethnicity^a^

	Total	White	South Asian	Black Caribbean	Black African
Characteristics	*N* = 444,418	*N* = 428,929	*N* = 8454	*N* = 3991	*N* = 3044
Age (IQR)	57.0 (50.0–63.0)	57.0 (50.0–63.0)	52.0 (45.0–59.0)	51.0 (46.0–57.0)	49.0 (44.0–56.0)
Male, *n* (%)	196,556 (44.2%)	189,260 (44.1%)	4325 (51.2%)	1446 (36.2%)	1525 (50.1%)
Systolic blood pressure (mm Hg)	137.8 ± 18.6	137.9 ± 18.6	134.6 ± 18.4	136.6 ± 18.5	138.4 ± 18.5
Diastolic blood pressure (mm Hg)	82.4 ± 10.1	82.4 ± 10.1	82.8 ± 10.0	84.3 ± 10.6	85.1 ± 10.7
Body mass index (kg/m^2^), *n* (%)					
18.5–24.9	149,622 (33.7%)	145,519 (33.9%)	2766 (32.7%)	845 (21.2%)	492 (16.2%)
< 18.5	2345 (0.5%)	2284 (0.5%)	54 (0.6%)	5 (0.1%)	2 (0.1%)
25.0–29.9	188,357 (42.4%)	181,679 (42.4%)	3776 (44.7%)	1649 (41.3%)	1253 (41.2%)
≥ 30	104,094 (23.4%)	99,447 (23.2%)	1858 (22.0%)	1492 (37.4%)	1297 (42.6%)
Household income, n(%)					
< 18,000	82,607 (18.6%)	78,381 (18.3%)	2231 (26.4%)	1068 (26.8%)	927 (30.5%)
18,000–30,999	127,545 (28.7%)	122,398 (28.5%)	2699 (31.9%)	1386 (34.7%)	1062 (34.9%)
31,000–51,999	127,815 (28.8%)	124,096 (28.9%)	1932 (22.9%)	1044 (26.2%)	743 (24.4%)
52,000–100,000	84,853 (19.1%)	82,935 (19.3%)	1203 (14.2%)	448 (11.2%)	267 (8.8%)
> 100,000	21,598 (4.9%)	21,119 (4.9%)	389 (4.6%)	45 (1.1%)	45 (1.5%)
Education, *n* (%)					
Less than high school	22,423 (5.0%)	21,521 (5.0%)	388 (4.6%)	241 (6.0%)	273 (9.0%)
High school or equivalent	224,478 (50.5%)	218,013 (50.8%)	3577 (42.3%)	2014 (50.5%)	874 (28.7%)
high school above	197,517 (44.4%)	189,395 (44.2%)	4489 (53.1%)	1736 (43.5%)	1897 (62.3%)
Townsend deprivation index, *n* (%)					
Quintile 1 (least deprived)	91,998 (20.7%)	90,972 (21.2%)	816 (9.7%)	140 (3.5%)	70 (2.3%)
Quintile 2	90,574 (20.4%)	89,416 (20.8%)	834 (9.9%)	222 (5.6%)	102 (3.4%)
Quintile 3	90,163 (20.3%)	88,335 (20.6%)	1239 (14.7%)	398 (10.0%)	191 (6.3%)
Quintile 4	88,438 (19.9%)	84,505 (19.7%)	2457 (29.1%)	998 (25.0%)	478 (15.7%)
Quintile 5 (most deprived)	83,245 (18.7%)	75,701 (17.6%)	3108 (36.8%)	2233 (56.0%)	2203 (72.4%)
LDL-cholesterol (mmol/L)	3.6 ± 0.8	3.6 ± 0.8	3.4 ± 0.8	3.3 ± 0.8	3.2 ± 0.8
HDL-cholesterol (mmol/L)	1.5 ± 0.4	1.5 ± 0.4	1.3 ± 0.3	1.5 ± 0.3	1.4 ± 0.3
Triglyceride (mmol/L)	1.7 ± 1.0	1.7 ± 1.0	1.9 ± 1.1	1.3 ± 0.7	1.3 ± 0.7
eGFR (mL/min/1.73m^2^)	95.8 ± 11.5	95.8 ± 11.4	100.3 ± 11.6	90.8 ± 13.1	93.4 ± 13.5
HbA1c (%, IQR)	5.4 (5.1–5.6)	5.4 (5.1–5.6)	5.8 (5.3–5.9)	5.7 (5.3–5.9)	5.7 (5.3–5.9)
Diabetes, *n* (%)	18,031 (4.1%)	16,234 (3.8%)	1156 (13.7%)	370 (9.3%)	271 (8.9%)
Antihypertensive medication, *n* (%)	43,732 (9.8%)	41,605 (9.7%)	849 (10.0%)	684 (17.1%)	594 (19.5%)
Cholesterol-lowering medication, *n* (%)	56,038 (12.6%)	53,459 (12.5%)	1693 (20.0%)	490 (12.3%)	396 (13.0%)
Drinking status, *n* (%)					
Never drinker	17,750 (4.0%)	13,236 (3.1%)	3336 (39.5%)	344 (8.6%)	834 (27.4%)
Former drinker	14,653 (3.3%)	13,831 (3.2%)	443 (5.2%)	156 (3.9%)	223 (7.3%)
Current drinker	412,015 (92.7%)	401,862 (93.7%)	4675 (55.3%)	3491 (87.5%)	1987 (65.3%)
Smoking status, *n* (%)					
Never	248,263 (55.9%)	236,684 (55.2%)	6621 (78.3%)	2539 (63.6%)	2419 (79.5%)
Current	46,044 (10.4%)	44,360 (10.3%)	813 (9.6%)	667 (16.7%)	204 (6.7%)
Previous	150,111 (33.8%)	147,885 (34.5%)	1020 (12.1%)	785 (19.7%)	421 (13.8%)
Physical activities (METmin/week), median (IQR)	2086.0 (990.0–3477.2)	2095.0 (993.0–3492.0)	1794.0 (772.5–3077.3)	2055.0 (990.0–3344.0)	1794.5 (833.5–3111.5)

Abbreviation: *BMI* Body mass index, *LDL-C* Low-density lipoprotein cholesterol, *HDL-C* High-density lipoprotein cholesterol, *eGFR* Estimated glomerular filtration rate, *IQR* Interquartile range, *SD* Standard deviation

^a^Data are presented as mean ± SD or *n* (%), unless otherwise indicated

**Fig. 1:**
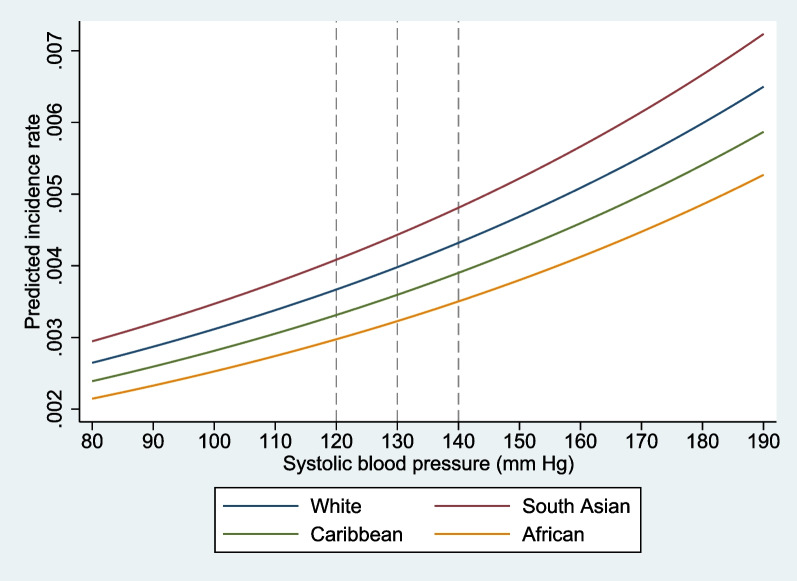
Predicted incidence rate of composite outcome of atherosclerotic cardiovascular disease, heart failure, and chronic kidney disease associated with different systolic blood pressure levels in four ethnic populations. The grey dashed line indicates the ESH recommended the optimal systolic blood pressure threshold of 120 mm Hg, the high normal threshold of 130 mm Hg, and the grade 1 hypertension threshold of 140 mm Hg. Incidence rates were adjusted for age, sex, BMI, income, education, drinking status, smoking status, diabetes status, hypertension medication, cholesterol-lowering medication, LDL-C, HDL-C, triglycerides, eGFR, Townsend deprivation index, and physical activities. Abbreviation: eGFR, estimated glomerular filtration rate; BMI, body mass index; LDL-C, low-density lipoprotein cholesterol; HDL-C, high-density lipoprotein cholesterol; ESH, European Society of Hypertension

After a median follow-up of 12.5 years (interquartile range, 11.7–13.2), 32,662 (7.4%) of the 444,418 participants had incident composite outcomes of atherosclerotic cardiovascular disease, heart failure, and chronic kidney disease. Additional file 1: Table S3 shows the number of cases and crude incidence rate among each ethnic population throughout the entire study period. Figure 1 displays the predicted incidence rates of the composite outcomes, plotted against SBP for different ethnic groups. After adjusting for age, sex, BMI, income, education, drinking status, smoking status, diabetes status, hypertension medication, cholesterol-lowering medication, LDL-C, HDL-C, triglycerides, eGFR, Townsend deprivation index, and physical activities, at any given level of SBP, the incidence rate of the composite outcome was the highest among Asian individuals, followed by White and Black Caribbean individuals, while it was the lowest among Black African population. For example, at SBP level of 120 mm Hg, the incidence rate was 4.20 (95% confidence interval (CI), 3.86–4.54) for South Asian, 3.67 (95% CI, 3.59–3.75) for White population, 3.23 (95% CI, 2.83–3.62) for Caribbean, and 3.00 (95% CI, 2.55–3.45) for African (Table 2).

**Table 2:** Ethnicity-specific thresholds of systolic blood pressure yielding risks equivalent to those associated with recommended blood pressure thresholds in the white population

	Incidence rate (95% CI) per 1000 person-years^a^				Risk-equivalent threshold (mm Hg)^b^		
ESH thresholds (mm Hg)^c^	White	South Asian	Black Caribbean	Black African	South Asian	Black Caribbean	Black African
120	3.67 (3.59,3.75)	4.20 (3.86,4.54)	3.23 (2.83,3.62)	3.00 (2.55,3.45)	103	136	145
130	3.98 (3.92,4.05)	4.56 (4.20,4.93)	3.50 (3.08,3.93)	3.25 (2.77,3.74)	113	146	155
140	4.32 (4.26,4.38)	4.95 (4.56,5.34)	3.80 (3.34,4.26)	3.53 (3.01,4.05)	123	156	165

Abbreviation: *IR* Incidence rate, *CI* Confidence interval, *eGFR* Estimated glomerular filtration rate, *BMI* Body mass index, *LDL-C* Low-density lipoprotein cholesterol, *HDL-C* High-density lipoprotein cholesterol, *ESH* European Society of Hypertension

^a^Predicted incidence rate was for the composite outcome of atherosclerotic cardiovascular disease, heart failure, and chronic kidney disease, adjusted for age, sex, BMI, income, education, drinking status, smoking status, diabetes status, hypertension medication, cholesterol-lowering medication, LDL-C, HDL-C, triglycerides, eGFR, Townsend deprivation index, and physical activities

^b^Risk-equivalence thresholds were rounded to the nearest whole number

^c^The ESH guidelines recommend the following systolic blood pressure categories: optimal (< 120 mm Hg), normal (120–129 mm Hg), high normal (130–139 mm Hg), and grade 1 hypertension (140–159 mm Hg)

To achieve an equivalent incidence of the composite outcomes observed in the White population at SBP levels of 140, 130, and 120 mmHg after adjusting for the full set of covariates, the corresponding SBP thresholds for South Asians were rounded to 123, 113, and 103 mmHg. For Black Caribbean individuals, the corresponding SBP thresholds were 156, 146, and 136 mmHg, and for Black African individuals were 165, 155, and 145 mmHg, respectively (Table 2). The risk-equivalent thresholds we identified after further adjustment of the duration of the residence in the UK were similar to previous results, although the thresholds were 12 mm Hg lower in the Black African populations compared to the pre-adjustment thresholds (Additional file 1: Table S4).

The thresholds obtained for the population untreated with antihypertensive medications were similar to those obtained for the whole population, while for the treated population, the thresholds were more extreme. The risk-equivalent thresholds corresponding to SBP levels of 140, 130, and 120 mmHg were 116, 106, and 96 mm Hg for South Asians, 176, 166, and 156 mm Hg for both Black Caribbean, and 175, 165, and 155 mm Hg for Black African **(**Table 3**)**. The risk-equivalent thresholds obtained among participants < 50 years showed the strongest deviation from the guideline-recommended thresholds, while thresholds for older participants were closer to the guideline thresholds (Additional file 1: Table S5). Thresholds also differed between males and females, with female thresholds closer to guideline recommendations (Additional file 1: Table S6).

**Table 3:** Ethnicity-specific thresholds of systolic blood pressure yielding risks equivalent to those associated with recommended thresholds in the white population with/without hypertension treatment

		Risk-equivalent threshold (mm Hg)^b^		
ESH thresholds (mm Hg)^c^	Incidence rate (95% CI) per 1000 person-years in White^a^	South Asian	Black Caribbean	Black African
**Treated**				
120	7.85 (7.38,8.33)	96	156	155
130	8.24 (7.86,8.61)	106	166	165
140	8.64 (8.34,8.94)	116	176	175
**Untreated**				
120	3.35 (3.27,3.43)	104	134	149
130	3.66 (3.59,3.72)	114	144	159
140	3.99 (3.93,4.05)	124	154	169

Abbreviation: *IR* Incidence rate, *CI* Confidence interval, *eGFR* Estimated glomerular filtration rate, *BMI* Body mass index, *LDL-C* Low-density lipoprotein cholesterol, *HDL-C* High-density lipoprotein cholesterol, *ESH* European Society of Hypertension

^a^Predicted incidence rate was for the composite outcome of atherosclerotic cardiovascular disease, heart failure, and chronic kidney disease, adjusted for age, sex, BMI, income, education, drinking status, smoking status, diabetes status, hypertension medication, cholesterol-lowering medication, LDL-C, HDL-C, triglycerides, eGFR, Townsend deprivation index, and physical activities

^b^Risk-equivalence thresholds were rounded to the nearest whole number

^c^The ESH guidelines recommend the following systolic blood pressure categories: optimal (< 120 mm Hg), normal (120–129 mm Hg), high normal (130–139 mm Hg), and grade 1 hypertension (140–159 mm Hg)

Grade 1 hypertension defined by ethnicity-specific threshold was significantly associated with the composite outcome in South Asian (Incidence rate ratio (IRR), 1.33 [95% CI, 1.03,1.73]) and Black African population (IRR, 1.70 [95% CI, 1.08,2.67]) while hypertension defined by guideline-recommended thresholds was significant only in South Asian (IRR, 1.29 [95% CI, 1.06,1.56]) (Table 4). The association was stronger using the ethnicity-specific thresholds than guideline-recommended thresholds in all ethnicity groups. Compared to guideline-recommended thresholds, ethnicity-specific thresholds resulted in higher population-attributable fractions (PAFs) of the composite outcome associated with grade 1 hypertension in South Asian (PAF, 21.5% vs. 11.3%) and Black African populations (PAF, 7.1% vs. 5.7%) (Additional file 1: Table S7).

**Table 4:** Association of grade 1 hypertension defined by ethnicity-specific thresholds vs. ESH thresholds with incident composite outcome

	IRR (95% CI)^a^	
Ethnicity	Ethnicity-specific threshold^b^	ESH threshold
South Asian	**1.33 (1.03,1.73)**^c^	**1.29 (1.06,1.56)**
Black Caribbean	1.26 (0.91,1.75)	1.21 (0.89,1.66)
Black African	**1.70 (1.08,2.67)**	1.11 (0.77,1.61)

Abbreviation: *IRR* Incidence rate ratio, *CI* Confidence interval, *eGFR* Estimated glomerular filtration rate, *BMI* Body mass index, *LDL-C* Low-density lipoprotein cholesterol, *HDL-C* High-density lipoprotein cholesterol, *ESH* European Society of Hypertension

^a^IRR was for the incidence rate associated with grade 1 hypertension for the composite outcome of atherosclerotic cardiovascular disease, heart failure, and chronic kidney disease, adjusted for age, sex, BMI, income, education, drinking status, smoking status, diabetes status, hypertension medication, cholesterol-lowering medication, LDL-C, HDL-C, triglycerides, eGFR, Townsend deprivation index, and physical activities

^b^Ethnicity-specific thresholds for grade 1 hypertension were as shown in Table 2

^c^Bolded numbers were statistically significant at the level of 0.05

## Discussion

In the prospective analysis of 444,418 participants in the UK Biobank without clinical CVD and chronic kidney disease, we found that at any given SBP, the predicted incidence rate of the composite outcome of atherosclerotic cardiovascular disease, heart failure, and chronic kidney disease was the highest in South Asian, followed by White, Black Caribbean, and Black African, after adjusting for traditional risk factors, behavior factors, and socioeconomic factors. For an equivalent risk of the composite outcomes observed in the White population at an SBP level of 140 mm Hg, we found a lower SBP threshold for South Asians (123 mm Hg) and higher thresholds for Black Caribbean (156 mm Hg) and Black African (165 mm Hg). Furthermore, we found that hypertension defined by ethnicity-specific thresholds was a stronger predictor and resulted in a larger population-attributable fraction for the composite outcomes in the South Asian and Black African populations compared to hypertension defined by the guideline-recommended thresholds.

Our findings of lower incidence rates of composite outcomes in Black Caribbean individuals and higher incidence rates in South Asians at any given SBP are consistent with previous studies in the UK and indicate that the guideline-recommended blood pressure thresholds applied universally to all ethnic groups might lead to imprecise risk estimation. A prospective study in the UK Biobank found a significantly higher risk of cardiovascular events in South Asians after adjusting for traditional cardiovascular risk factors that included blood pressure, sociodemographic, lifestyle, and environmental factors, and lower risk in Black Caribbean and Black African individuals, although not statistically significant after adjustment [32]. A study of the SABRE (Southall and Brent Revisited) cohort [4] and another study involving over one million participants recruited from electronic health records in the UK [1] both revealed a noteworthy pattern. They demonstrated that, even after adjusting for conventional risk factors, the risk of CHD was significantly elevated in South Asians and lowered in African Caribbeans compared to White populations. Traditional cardiometabolic, genetic and epigenetic, lifestyle, socioeconomic factors, environmental factors, access to care, and their interactions could account for these disparities, necessitating further research.

According to the current ESH guidelines for managing arterial hypertension, adults diagnosed with grade 1 hypertension (SBP 140–159 mm Hg) and who are at low to moderate risk without hypertension-mediated organ damage are advised to initiate pharmacological interventions if lifestyle modifications fail to reduce blood pressure effectively. The recommended treatment target for most hypertensive patients is to achieve an SBP of less than 140 mm Hg, regardless of ethnicity [7]. However, these thresholds may not be fully applicable to the heterogeneous European populations, given that the recommendations were based on trials that were underpowered to evaluate differential treatment effects across ethnic groups [33], and people of different ethnic backgrounds exhibit different prevalence of hypertension, different pathophysiological responses to hypertension, and differential pharmacodynamic responses to antihypertensive medications [5]. In fact, current guidelines urge for more research in the European populations addressing the optimal hypertension management modality in ethnic subgroups, as such evidence was mostly extrapolated from studies in the USA and may have limited applications to ethnic subgroups in Europe, considering the genetic and socioeconomic differences [5, 7]. To the best of our knowledge, our study was the first to derive risk-equivalence SBP thresholds in non-White populations in an attempt to address the lack of optimal blood pressure thresholds for triggering actions in non-White populations.

Our study revealed a lower SBP threshold of 124 mm Hg for the untreated and 116 mm Hg for the treated South Asians to have an equivalent risk to that of the White population at an SBP of 140 mm Hg, suggesting that a lower threshold for initiating interventions and a lower treatment target may be necessary for South Asians. Meta-analyses and systemic reviews of the trials supporting current ESH guidelines generally endorse the beneficial effects of initiating treatments [34] and targeting more aggressive blood pressure thresholds [35] for high cardiovascular-risk patients, such as those with established CVDs, with blood pressure at a high normal range (SBP 130–140 mm Hg). In contrast, due to the inconsistent results for the benefits of treating low-to-moderate risk patients with SBP < 140 mm Hg, current guidelines refrain from recommending antihypertensive treatment for such patients [7]. However, our study demonstrated that the ethnicity of South Asia could be a risk enhancer that places them in the high-risk group, necessitating prompt interventions even when SBP was within the normal range and in the absence of established CVDs. South Asians tend to have a lower level of SBP [36] but more severe forms of cardiovascular complications and organ damage than the White populations [37]. Moreover, a previous study reported a stronger association between SBP and stroke in South Asians compared to Whites [10], and residue cardiovascular risk still exists even when blood pressure is treated to the normal range [38]. Thus, the current ethnicity-blind hypertension thresholds of 140 mm Hg will provide a false sense of security for South Asians, preventing them from taking prompt actions to halt the progression into more serious complications. This is especially noteworthy since nearly 50% of the global disease burden attributed to blood pressure disorders is found among individuals with an SBP < 140 mm Hg [26], and we found that 21.5% of the cardiorenal complications among South Asians could be eliminated if using ethnicity-specific hypertension thresholds, in contrast to 11.3% if using guideline-recommended thresholds.

On the contrary, we found higher risk-equivalent SBP thresholds in untreated Black Caribbean (154 mm Hg) and untreated Black African (169 mm Hg) for an SBP of 140 mm Hg in the White population, and the thresholds were even higher among the treated population. While previous studies have shown that the Black population tends to have a higher prevalence of hypertension, a higher prevalence of treated hypertension [36], but worse blood pressure control than the White population [39], our results suggest that the less controlled blood pressure might not confer higher risks for Black individuals and the current hypertension threshold of SBP 140 mm Hg may overestimate the risks of adverse outcomes for them. In fact, we demonstrated that SBP > 140 mm Hg was not associated with a significantly higher risk of cardiorenal complications among Black participants. As guidelines are inconclusive regarding the benefits of pharmacological treatments for low-risk populations [5, 7, 31], and larger numbers are needed to treat to prevent one cardiovascular event in the short run for low-risk individuals [31], our study raises the question of whether hypertension thresholds should be raised for the Black population in the UK.

With increasing age, the evidence supporting ethnicity-specific cutoffs becomes weaker. We did not find higher risk-equivalence cutoffs for Black African populations aged 60 and above than the guideline-recommended cutoffs, which could be due to the aging process affecting the association of blood pressure with adverse outcomes more significantly than ethnicity. In fact, previous studies have found reduced disparities in stroke incidence across ethnic groups with aging and complete elimination of disparities at the age of 85 [40, 41]. SBP tends to increase with age, and findings from the Framingham Heart Study suggest that around 90% of adults without hypertension at ages 55 or 65 will develop hypertension during their life [42], indicating that the need for ethnicity-specific SBP cutoffs among older adults may be unnecessary.

Our study has several limitations. Firstly, although we found higher risk-equivalent SBP thresholds for the Black populations, the association between SBP and cardiovascular and renal complications has been reported to be continuous across levels of SBP. A follow-up study of the SPRINT trial in the US did not find significant racial-ethnic differences in the beneficial effect of intensive blood pressure control [33]. Therefore, further research is required to corroborate the higher SBP thresholds for triggering interventions in Black Caribbean and Black African populations, especially in the context of the UK. This is particularly important given that there is evidence that the degree and phenotype of hypertension-mediated target organ damage might be more severe in the Black population compared with the White population at any given SBP level in the US [12]. Secondly, the risk-equivalent thresholds we derived were based on composite outcomes of atherosclerotic cardiovascular disease, heart failure, and chronic kidney disease. The thresholds might differ if different outcomes or components of composite outcomes were used. Nonetheless, our composite outcomes encompassed the most important hypertension-related sequelae. Thirdly, while we have rigorously controlled for an extensive array of potential confounders, including sociodemographic factors, lifestyle characteristics, clinical attributes, and biomarkers, as well as duration of residence in the UK, the possibility of residual confounding remains. For example, there might be differential healthcare-seeking behavior among different ethnic groups, which requires further research and could lead to outcome ascertainment bias for less acute outcomes. Fourthly, while the UK Biobank was broadly representative of the general UK population in terms of sociodemographic characteristics and the effect estimates of the risk factors were not biased compared to population-based studies [43], it was not specifically designed to accurately represent the broader ethnic minority population or ethnic minorities in other regions. Moreover, although we included the three largest populations living currently in the UK, we cannot analyze other ethnic groups due to a limited number of participants or lack of categorization for other ethnic groups. It is worth noting that the potential "healthy volunteer" bias in the UK Biobank might result in a lower absolute risk of adverse outcomes and lower PAFs associated with hypertension. However, this reduction is unlikely to vary significantly across ethnic groups, thus minimizing the potential for bias in our estimates of ethnic disparities. Fifthly, blood pressure measurements were taken by trained staff in a standardized and uniform manner for participants in the UK Biobank. Therefore, we are not certain that our derived thresholds can apply to populations in other countries or future generations with different measuring schemes. Lastly, obtaining average blood pressure measurements from 2 to 3 separate occasions can minimize random error and provide more accurate blood pressure estimations to avoid regression dilution bias, although differential assessment of the blood pressure across ethnic groups was unlikely to occur in our study.

Despite the limitations, the study had several strengths. It involved a large cohort with sufficient participants from the four ethnic populations, enabling us to reliably derive ethnicity-specific cutoffs in each ethnic group and subgroups by age, sex, and treatment status. It also had a prospective design with a long follow-up period for temporality and event accumulation to allow us to improve the precision of our estimates. Outcomes ascertainment through linkage to hospital in-patient records and ICD-10 codes helps to minimize the chances of incomplete ascertainment and misclassification of outcomes. Moreover, the blood pressure measurements and other covariates were standardized and uniform, improving our study’s internal validity. We adjusted for various potential confounders, encompassing sociodemographic factors, lifestyle characteristics, clinical attributes, and biomarkers. Moreover, we adjusted for area-level measurements of deprivation to separate the effect of ethnicity from socioeconomic status and more accurately estimate ethnic disparities in cardiorenal complications.

## Conclusions

The choice of hypertension thresholds has a significant influence not only on the decision to initiate pharmacological interventions but also on raising public awareness for prompt lifestyle modifications to halt the progression into cardiovascular and renal complications. Our findings suggest that the risks of cardiovascular and renal complications differ among ethnic groups at any given level of SBP. Thus, current guidelines recommended blood pressure thresholds may overestimate the risks of adverse outcomes for Black Caribbeans and Black Africans while underestimating the risks for South Asians. In contrast, risk-equivalent ethnicity-specific blood pressure thresholds may help to characterize the risk of adverse outcomes more accurately in ethnic minority groups, thus guiding the efficient allocation of medical resources towards the goal of resolving the ethnic disparities in hypertension management. Future research should investigate whether there is improved cost-effectiveness of initiating interventions based on ethnicity-specific SBP thresholds in preventing cardiovascular and renal complications.

### Supplementary Information

**Additional file 1: Figure S1.** Flowchart for inclusion in the analytic sample of participants in the UK biobank. **Table S1**. Manual blood pressure measurement distribution and values compared to automated measurements. **Table S2**. Outcome definitions and corresponding ICD 9/10 codes. **Table S3**. Number of cases and the crude incidence rate for the composite outcomes. **Table S4**. Ethnicity-specific thresholds of systolic blood pressure after adjusting for immigration history. **Table S5**. Ethnicity-specific thresholds of systolic blood pressure in different age groups. **Table S6**. Ethnicity-specific thresholds of systolic blood pressure by sex groups. **Table S7**. Population attributable fraction of the composite outcome associated with grade 1 hypertension defined by ethnicity-specific thresholds vs. ESH-recommended thresholds.

## Data Availability

This research has been conducted using data from UK Biobank, a major biomedical database. UKF biobank data can be required from https://www.ukbiobank.ac.uk/.

## Abbreviations

CHD: Coronary heart disease
BP: Blood pressure
SBP: Systolic blood pressure
DBP: Diastolic blood pressure
ESH: European Association of Hypertension
BMI: Body mass index
LDL-C: Low-density lipoprotein cholesterol
HDL-C: High-density lipoprotein cholesterol
NICE: National Institute of Health and Care Excellence
eGFR: Estimated glomerular filtration rate
CKD: Chronic kidney disease
ASCVD: Atherosclerotic cardiovascular disease
HF: Heart failure
PAF: Population attributable fraction
CI: Confidence interval
IRR: Incidence rate ratio
