# Supplementary material for: Ethnicity-specific blood pressure thresholds based on cardiovascular and renal complications: a prospective study in the UK Biobank
Source: BMC Med. 2024 Feb 5;22:54. doi: 10.1186/s12916-024-03259-5 (PMC10845677; doi:10.1186/s12916-024-03259-5)
Supplement: Supplementary file 1 — Additional file 1: Figure S1. Flowchart for inclusion in the analytic sample of participants in the UK biobank. Table S1. Manual blood pressure measurement distribution and values compared to automated measurements. Table S2. Outcome definitions and corresponding ICD 9/10 codes. Table S3. Number of cases and the crude incidence rate for the composite outcomes. Table S4. Ethnicity-specific thresholds of systolic blood pressure after adjusting for immigration history. Table S5. Ethnicity-specific thresholds of systolic blood pressure in different age groups. Table S6. Ethnicity-specific thresholds of systolic blood pressure by sex groups. Table S7. Population attributable fraction of the composite outcome associated with grade 1 hypertension defined by ethnicity-specific thresholds vs. ESH-recommended thresholds. [file 12916_2024_3259_MOESM1_ESM.doc]

**Ethnicity-specific blood pressure thresholds based on cardiovascular and renal complications: a prospective study in the UK Biobank**

**Authors:** Donghan Su, Huanhuan Yang, Zekun Chen, Yuhao Kong, Xiaona Na, Queran Lin, Ai Zhao, Yan Zheng, Yanan Ma, Xiaoyu Li, Zhihui Li

**Corresponding authors:**

Dr. Zhihui Li

Vanke School of Public Health, Tsinghua University, Beijing, China

Institute for Healthy China, Tsinghua University, Beijing, China

Department of Social and Behavioral Sciences, Harvard T.H. Chan School of Public Health, Boston, MA, 02115, USA.

zhihuili@mail.tsinghua.edu.cn

Figure S1: Flowchart for inclusion in the analytic sample of participants in the UK Biobank.

Table S1. Manual blood pressure measurement distribution and values compared to automated measurements

|  | Overall | White | South Asian | Black Caribbean | Black African |
| --- | --- | --- | --- | --- | --- |
| Manual measurements, n(%) | 25,621 (5.77%) | 25,317 (5.90%) | 217 (2.57%) | 35 (0.88%) | 52 (1.71%) |
| Manual SBP measurements, mm Hg, mean (IQR) | 137.5 (126.0-150.5) | 139.0 (126.0-150.5) | 134.7 (122.0-146.0) | 133.8 (119.0-149.5) | 135.3 (124.3-145.5) |
| Automated SBP measurements, mm Hg, mean (IQR) | 136.0 (124.5-149.0) | 138.0 (124.5-149.5) | 134.7 (122.0-146.0) | 136.7 (123.0-148.5) | 138.4 (125.0-150.0) |

Abbreviation: SBP, systolic blood pressure; IQR, interquartile range

Table S2: Outcome definitions and corresponding ICD 9/10 codes

| Outcome | | ICD-9 | ICD-10 |
| --- | --- | --- | --- |
|  | Myocardial Infarction | 410-412, 429.79 | I21-I23, i24.1, I225.2 |
|  | Stroke | 430, 431, 432, 433, 434, 436,437, 438 | I60, I61, I63, and I64 |
|  | Chronic kidney disease | 5859 | N180-N185 |
|  | Heart failure | 428.x | I50.x |
| Other CVD causes |  |  |  |
|  | Stable/unstable angina | 413.1, 413.9,411.1, 411.81, 411.89 | I20.0, I24.0, I24.8, I24.9, I20.1, I20.8, I20.9 |
|  | peripheral vascular disease | 250.6x, 440.2x, 443.1, 443.8, 443.9 | I73.x, I74.3, I74.4, I74.5 |
|  | Arrhythmia and conduction disorder | 426.x, 427.x | ICD-10: I44.x, I48.x |

Abbreviation: CVD, cardiovascular disease; ICD, International Classification of Disease

Table S3. Number of cases and the crude incidence rate for the composite outcomes*

| Ethnic groups | Cases | Incidence rate (95% CI) per 1000 person-years |
| --- | --- | --- |
| White | 31,552 | 6.028 (5.962,6.095) |
| South Asian | 660 | 6.583 (6.099,7.105) |
| Black Caribbean | 270 | 5.711 (5.069,6.435) |
| Black African | 180 | 4.958 (4.284,5.738) |

*The composite outcome included atherosclerotic cardiovascular disease, heart failure, and chronic kidney disease.

Abbreviation: CI, confidence interval

Table S4. Ethnicity-specific thresholds of systolic blood pressure after adjusting for immigration history*

|  | Incidence rate (95% CI) per 1000 person-years | Risk-equivalent threshold (mm Hg)^‡^ | | |
| --- | --- | --- | --- | --- |
| ESH thresholds (mm Hg)^†^ | White | South Asian | Black Caribbean | Black African |
| 120 | 3.662 (3.584,3.739) | 99 | 135 | 133 |
| 130 | 3.973 (3.908,4.038) | 109 | 145 | 143 |
| 140 | 4.311 (4.248,4.374) | 119 | 155 | 153 |

^*^Predicted incidence rate was for the composite outcome of atherosclerotic cardiovascular disease, heart failure, and chronic kidney disease, adjusted for age, sex, BMI, income, education, drinking status, smoking status, history of diabetes, hypertension medication, cholesterol-lowering medication, LDL-C, HDL-C, triglycerides, eGFR, Townsend deprivation index, physical activities, and the duration of residence in the UK.

^†^The ESH guidelines recommend the following systolic blood pressure categories: optimal (<120 mm Hg), normal (120-129 mm Hg), high normal (130-139 mm Hg), and grade 1 hypertension (140-159 mm Hg).

^‡^Risk-equivalence thresholds were rounded to the nearest whole number.

Abbreviation: CI, confidence interval; eGFR, estimated glomerular filtration rate; BMI, body mass index; LDL-C, low-density lipoprotein cholesterol; HDL-C, high-density lipoprotein cholesterol; ESH, European Society of Hypertension

Table S5. Ethnicity-specific thresholds of systolic blood pressure in different age groups

|  |  | Risk-equivalent threshold (mm Hg)^†^ | | |
| --- | --- | --- | --- | --- |
| ESH Threshold (mm Hg) | Incidence rate (95% CI) per 1000 person-years in White* | South Asian | Black Caribbean | Black African |
| **Age < 50** |  |  |  |  |
| 120 | 1.41 (1.33,1.50) | 103 | 152 | 183 |
|  |  |  |  |  |
| 130 | 1.51 (1.44,1.58) | 113 | 162 | 193 |
| 140 | 1.61 (1.52,1.71) | 123 | 172 | 203 |
|  | | | | |
| **Age 50-60** |  |  | | |
| 120 | 2.96 (2.84,3.08) | 108 | 131 | 169 |
| 130 | 3.30 (3.20,3.39) | 118 | 141 | 179 |
| 140 | 3.67 (3.8,3.77) | 128 | 151 | 189 |
|  | | | | |
| **Age ≥ 60** |  |  | | |
| 120 | 6.70 (6.51,6.88) | 115 | 135 | 118 |
| 130 | 7.41 (7.25,7.57) | 125 | 145 | 128 |
| 140 | 8.20 (8.07,8.33) | 135 | 155 | 138 |

*Predicted incidence rate was for the composite outcome of atherosclerotic cardiovascular disease, heart failure, and chronic kidney disease, adjusted for age, sex, BMI, income, education, drinking status, smoking status, history of diabetes, hypertension medication, cholesterol-lowering medication, LDL-C, HDL-C, triglycerides, eGFR, Townsend deprivation index, and physical activities.

^†^Risk-equivalence thresholds were rounded to the nearest whole number.

Abbreviation: CI, confidence interval; eGFR, estimated glomerular filtration rate; BMI, body mass index; LDL-C, low-density lipoprotein cholesterol; HDL-C, high-density lipoprotein cholesterol; ESH, European Society of Hypertension

Table S6. Ethnicity-specific thresholds of systolic blood pressure by sex groups

|  |  | Risk-equivalent threshold (mm Hg)^†^ | | |
| --- | --- | --- | --- | --- |
| ESH Threshold (mm Hg) | Incidence Rate (95% CI) per 1000 person-years in White* | South Asian | Black Caribbean | Black African |
| **Female** |  |  |  |  |
| 120 | 2.68 (2.60,2.76) | 114 | 121 | 124 |
| 130 | 2.92 (2.85,2.99) | 124 | 131 | 134 |
| 140 | 3.19 (3.12,3.27) | 134 | 141 | 144 |
|  |  |  |  |  |
| **Male** |  |  |  |  |
| 120 | 5.2 (5.04,5.36) | 93 | 158 | 161 |
| 130 | 5.60 (5.47, 5.72) | 103 | 168 | 171 |
| 140 | 6.03 (5.92,6.134) | 113 | 178 | 181 |

*Predicted incidence rate was for the composite outcome of atherosclerotic cardiovascular disease, heart failure, and chronic kidney disease, adjusted for age, sex, BMI, income, education, drinking status, smoking status, history of diabetes, hypertension medication, cholesterol-lowering medication, LDL-C, HDL-C, triglycerides, eGFR, Townsend deprivation index, and physical activities.

^†^Risk-equivalence thresholds were rounded to the nearest whole number.

Abbreviation: CI, confidence interval; eGFR, estimated glomerular filtration rate; BMI, body mass index; LDL-C, low-density lipoprotein cholesterol; HDL-C, high-density lipoprotein cholesterol; ESH, European Society of Hypertension

Table S7: Population attributable fraction of the composite outcome associated with grade 1 hypertension defined by ethnicity-specific thresholds vs. ESH recommended thresholds

|  | PAF % (95% CI)* | |
| --- | --- | --- |
| Ethnicity | Ethnicity-specific threshold | ESH threshold |
| South Asian | **21.5 (2.4,36.9)** | **11.3 (2.6,19.1)** |
| Black Caribbean | 5.5 (-2.8,13.2) | 10.0 (-6.9,24.3) |
| Black African | **7.1 (0.20,14.0)** | 5.7 (-16.2,23.5) |

*PAF represents the proportion of the composite outcome of atherosclerotic cardiovascular disease, heart failure, and chronic kidney disease that might be eliminated by eliminating grade 1 hypertension defined by ethnicity-specific thresholds as shown in Table 2 vs. ESH recommended thresholds, holding other variables constant, including age, sex, BMI, income, education, drinking status, smoking status, history of diabetes, hypertension medication, cholesterol-lowering medication, LDL-C, HDL-C, triglycerides, eGFR, Townsend deprivation index, and physical activities.

Abbreviations: PAF, population attributable fraction; CI, confidence interval; eGFR, estimated glomerular filtration rate; BMI, body mass index; LDL-C, low-density lipoprotein cholesterol; HDL-C, high-density lipoprotein cholesterol; ESH, European Society of Hypertension
